# TRPML Cation Channels in Inflammation and Immunity

**DOI:** 10.3389/fimmu.2020.00225

**Published:** 2020-02-28

**Authors:** Barbara Spix, Yu-Kai Chao, Carla Abrahamian, Cheng-Chang Chen, Christian Grimm

**Affiliations:** ^1^Faculty of Medicine, Walther Straub Institute of Pharmacology and Toxicology, Ludwig-Maximilians-Universität, Munich, Germany; ^2^Department of Pharmacy, Center for Drug Research, Ludwig-Maximilians-Universität, Munich, Germany

**Keywords:** immune system, immune cells, TRPML cation channels, mucolipin, lysosome

## Abstract

**Background:** In 1883, Ilya Mechnikov discovered phagocytes and established the concept of phagocytosis by macrophages. In 1908, he was awarded the Nobel Prize in Physiology/Medicine for his findings, which laid the foundations for today's understanding of the innate immune response. Only in the 1960s, Max Cooper and Robert Good significantly advanced our understanding of the immune system by demonstrating that B- and T-cells cooperate to regulate the adaptive immune response. Both, innate and adaptive immune response are essential to effectively protect the individual against infectious agents, such as viruses, bacterial or insect toxins, or allergens. Innate immune responses occur rapidly upon exposure to noxious or infectious agents or organisms, in contrast to the adaptive immune system that needs days rather than hours to develop and acts primarily on the basis of antigen-specific receptors expressed on the surface of B- and T-lymphocytes. In recent years, it has become evident that endosomes and lysosomes are involved in many aspects of immune cell function, such as phagocytosis, antigen presentation and processing by antigen-presenting cells, release of proinflammatory mediators, e.g., by mast cells, or secretion of the pore-forming protein perforin by cytotoxic T lymphocytes. Several lysosomal storage disorders (LSDs) have been associated with defects in immune system function or immune system hyperactivity, such as Gaucher, Fabry, or Niemann-Pick type C1 disease, mucopolysaccharidoses (MPS), gangliosidosis, or juvenile neuronal ceroid lipofuscinosis (JNCL). Beside accumulating evidence on the importance of endolysosomes in immune cell function, recent results suggest direct roles of endolysosomal ion channels, such as the TRPML channels (mucolipins), which are members of the transient receptor potential (TRP) superfamily of non-selective cation channels, for different aspects of immune cell function. The aim of this review is to discuss the current knowledge about the roles of TRPML channels in inflammation and immunity, and to assess their potential as drug targets to influence immune cell functions.

**Advances:** Examples of recently established roles of TRPML channels in immune system function and immune response include the TRPML1-mediated modulation of secretory lysosomes, granzyme B content, and tuning of effector function in NK cells, TRPML1-dependent directional dendritic cell (DC) migration and DC chemotaxis, and the role of TRPML2 in chemokine release from LPS-stimulated macrophages.

**Outlook:** Although our understanding of the functional roles of TRPML channels in inflammation and immunity is still in its infancy, a few interesting findings have been made in the past years, encouraging further and more detailed work on the role of TRPMLs, e.g., in intracellular trafficking and release of chemokines, cytokines, or granzyme B, or in phagocytosis and bacterial toxin and virus trafficking through the endolysosomal machinery.

## Introduction

**Figure 1:**
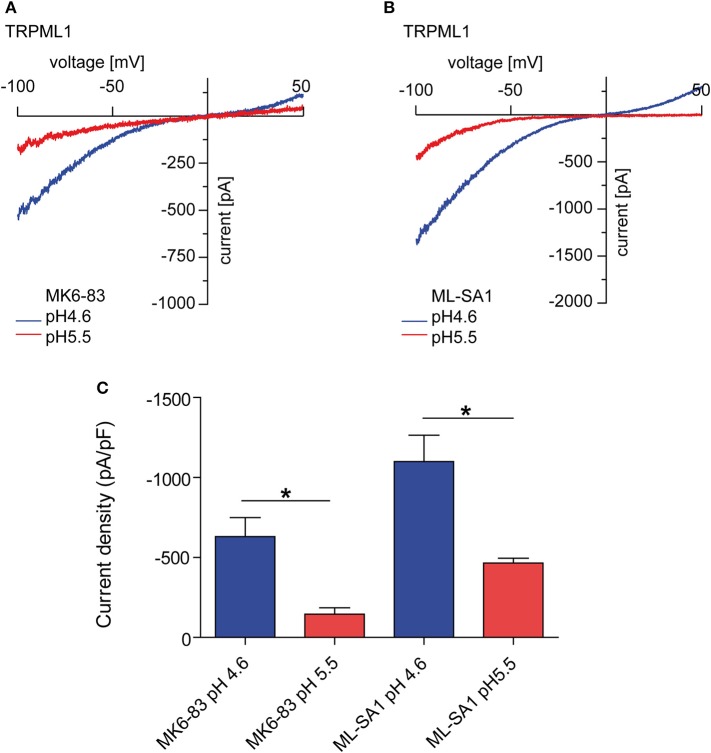
Endolysosomal patch-clamp experiments showing TRPML1 currents induced by MK6-83 and ML-SA1 (10 μM, each) at different luminal pH. **(A)** Representative MK6-83 elicited currents from TRPML1 expressing HEK293 cells at pH 4.6 or pH 5.5. **(B)** Representative ML-SA1 elicited currents from TRPML1 expressing HEK293 cells at pH 4.6 or 5.5. **(C)** Statistical summary of data as shown in **(A,B)**. Shown are average current densities at −100 mV (mean ± SEM). In all statistical analyses, the mean values of independent experiments (*n* > 4, each) are shown as indicated. *p < 0.05, Student's *t*-test, unpaired.

TRP channels are a very diverse and heterogenous group of cation channels. With few exceptions, the majority of them are expressed at the plasma membrane. One subfamily of the TRP channels, the mucolipin or TRPML/MCOLN subfamily comprises three members in mammalian genomes, TRPML1, 2, and 3 which are all found to be expressed in the endolysosomal system, i.e., in early and late endosomes, in recycling endosomes and in lysosomes to various degrees. All three channels are regulated by the phosphoinositide PI(3,5)P_2_, a major component of endolysosomal membranes and by luminal pH (1, 3–5). Thus, TRPML1 is most active at highly acidic pH as found in lysosomes while TRPML2 and TRPML3 are more active at higher pH as it occurs in early and recycling endosomes (1, 3–5). TRPML1 activity already decreases under mild acidic conditions (pH 5.5; Figure 1) and is lowest at neutral conditions (4). A recent hypothesis suggested that elevated lysosomal pH may hyperactivate the TRPML1 calcium channel (2), is not supported by endolysosomal patch-clamp evidence (Figure 1).

**Figure 2:**
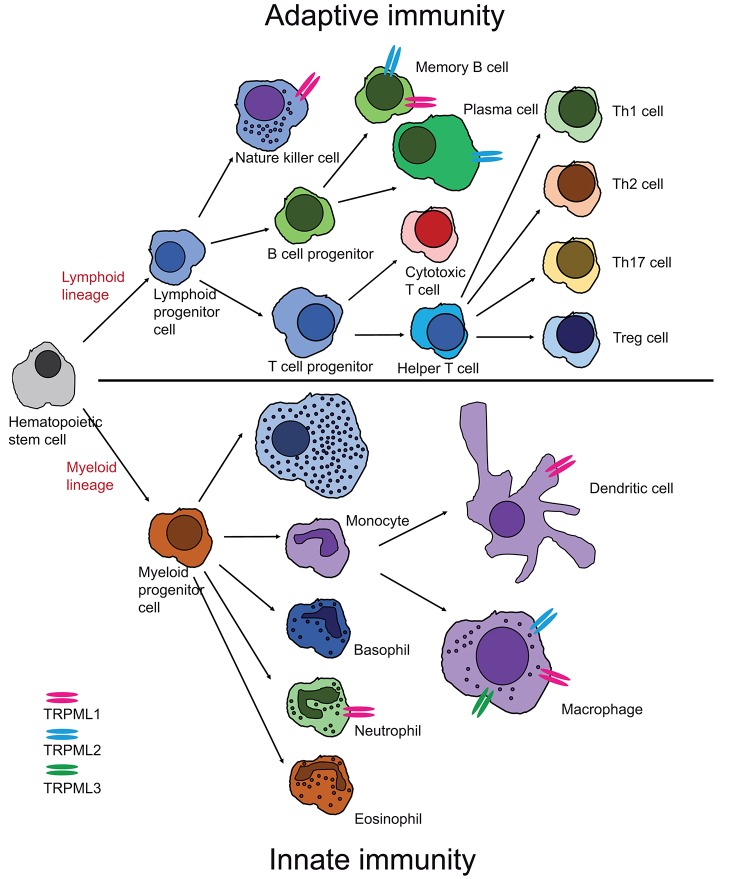
Cartoon showing expression of TRPML channels in different cells of the innate and adaptive immune system. Expression has either been confirmed by direct patch-clamp experimentation of endo-lysosomes isolated from these cells or by other published experimental evidence as outlined in chapter I and II.

Several functional roles have been proposed for TRPML1, e.g., in gastric acid secretion by parietal cells (6, 7), as a ROS sensor in lysosomes to regulate autophagy (8, 9), as an autophagy regulator through calcineurin and TFEB (10), or in membrane repair, e.g., repair of the sarcolemma to prevent muscular dystrophy (11). Loss or dysfunction of TRPML1 causes the rare lysosomal storage disorder mucolipidosis type IV, major hallmarks of which are severe neuro- and retinal degeneration, mental and psychomotor retardation, hypotonia, achlorhydria, and premature death. The observation that macromolecules, e.g., certain lipids (sphingolipids, phospholipids) and mucopolysaccharides as well as metals like iron or zinc accumulate in patient cells suggested roles for TRPML1 in metal cation release, in addition to the release of calcium and other cations from lysosomes as well as a critical function for the overall integrity of lysosomes including their roles in intracellular trafficking, fission/fusion and autophagy. While most research has focused on TRPML1, due to its clear association with human disease, function and pathophysiological relevance of the related channels TRPML2 and TRPML3 are less well-understood, with no links to human (genetic) disease so far. In mice, gain-of-function variants of TRPML3 have been shown to cause deafness, circling behavior, and coat color dilution due to the loss of inner ear hair cells and melanocytes following intracellular calcium overload. (12–16). TRPML2^−/−^ and TRPML3^−/−^ mice are viable and according to genomic databases, human TRPML2 and TRPML3 knockouts do also exist. Nothing however is known about health issues they may have. In contrast to the ubiquitously expressed TRPML1, the expression of TRPML2 and TRPML3 is restricted to certain cell types including a number of immune cells. Recently, several roles of immune cell function could be linked to TRPML channels, e.g., a role of TRPML1 for the phagocytosis of large particles (17), the migration of dendritic cells (18), or for the tuning of the functional potential in self-KIR+ natural killer (NK) cells (19) while TRPML2 was found to play a role in chemokine and cytokine secretion by macrophages (5, 20). In the following, the current state of knowledge on TRPML channel expression and function in different immune cells shall be discussed (Figure 2).

## Chapter I: Innate Immune System

### Macrophages

Macrophages are phagocytic cells. They express receptors on their surface called pattern recognition receptors (PRRs), which are able to detect varying molecular structures of microbes, referred to as pathogen-associated molecular pattern (PAMP). One example for a PRR are the toll-like receptors (TLRs). Sun et al. (20) have recently discovered that activation of these receptors leads to a strong increase of both mRNA and protein levels of the TRPML2 channel. They tested different TLR activators, including LPS (a component of bacteria that activates TLR4), zymosan A (a component of fungi that activates TLR2), loxoribine which activates TLR7, and resiquimod (R848) which activates TLR7/8. The latter two are involved in recognizing viruses. In all cases and both in primary (murine microglia) and cultured cells (RAW 264.7) an upregulation of TRPML2 was found, suggesting that the TRPML2 channel is involved in the host defense against different pathogens like bacteria, viruses and fungi.

The binding of such particles to the PRRs or the binding of IgG-opsonized particles to the FCγ-receptor trigger an important process in the innate immune response: phagocytosis. During this process the particle is surrounded by the macrophage with the help of extensions of the cytoplasm (i.e., pseudopods) and is completely enclosed with membrane in structures called phagosomes. These phagosomes mature by fusing with lysosomes into phagolysosomes, which have an acidic luminal pH and contain hydrolytic enzymes to break down the engulfed particles. Samie et al. (17) have first shown that TRPML1 regulates the ingestion of large particles by providing the membrane for the cell surface, necessary for phagosome formation. They suggested the following cascade of events and signaling pathways: after particle binding of the phagocytic cell the endolysosomal PI-5 kinase, PIKfyve is stimulated and phosphorylates PI(3)P to form PI(3,5)P_2_, which is then present in sufficient amounts to activate the TRPML1 channel in lysosomes. The resulting Ca^2+^ release induces lysosomal exocytosis at the site of the formation of the phagocytic cup. Such fusion events with the plasma membrane increase the surface area of the phagocytosing macrophage which is essential for the engulfment of large particles. Thus, TRPML1 plays a crucial role in the initial steps of phagocytosis and enables the innate immune system to eliminate large particles quickly (17). However, the TRPML1 channel does not only seem to be involved in phagosome formation but also in phagosome maturation, i.e., the fusion process of phagosomes with lysosomes. This was proposed by Dayam et al. (21) who analyzed phagocytosis in TRPML1-silenced or PIKfyve-inhibited cells in which the phagolysosomal biogenesis was impaired, because the phagosomes and lysosomes were not able to fuse after docking. The phagolysosome maturation could be rescued by Ca^2+^ ionophores like ionomycin. This suggested that both PIKfyve and TRPML1, more specifically the TRPML1-mediated Ca^2+^ release from lysosomes, were key mediators in phagosome maturation. PIKfyve would presumably act upstream of TRPML1, since it produces the endogenous TRPML1 activator PI(3,5)P_2_ (21). Impaired ability to eliminate the ingested bacteria was also observed in PIKfyve and TRPML1 deficient macrophages. This is in line with previous findings showing reduced levels of cathepsin D, a common lysosomal enzyme, in phagosomes of PIKfyve inhibited cells (22). Mycobacteria for example, interfere with the PI(3)P metabolism and thereby induce an impaired phagosome maturation, enabling them to prevent degradation through fusion with lysosomes (23).

Furthermore, TRPML1 has been found to enhance the degradative function of lysosomes during phagocytosis through TFEB (24). Gray et al. speculated that the protein phosphatase calcineurin, activated by the Ca^2+^ release through TRPML1, dephosphorylates TFEB to induce its translocation to the nucleus. There, it acts as a transcription factor for the lysosomal enzyme cathepsin D and the H subunit of the V-ATPase, which regulates the acidic and hydrolytic environment in the phagolysosomes. This enhances the degradation ability of the existing lysosomes, so that bacteria are eliminated more efficiently in the phagolysosomes. They showed this for opsonic phagocytosis mediated by the FCγ-receptor and for non-opsonic phagocytosis as well. Hence, the TRPML1 channel may not only be necessary for the initial steps of phagocytosis but also for later steps in the phagocytosis process like phagosome maturation and efficient degradation of engulfed particles. This is important, in particular when large numbers of bacteria are present.

Cathepsins and their proteolytic functions are essential for the degradation of bacteria captured in lysosomes during phagocytosis. The role of different cathepsins has been studied in great detail. Cathepsin D, besides its degradative capacity, may also induce bacterial killing by activating apoptosis in alveolar macrophages after take-up of pneumococci by phagosomes (25). Mice lacking cathepsin E are more sensitive to infections by Staphylococcus aureus and Porphyromonas gingivalis (26). Qi et al. (27) detected a role of cathepsin B in the defense of macrophages against Francisella novicida. Thus, bone marrow-derived macrophages (BMDM) from cathepsin B deficient mice were able to clear bacteria more efficiently and these mice were protected from lethality (27) which may be due to the down-regulation of mTOR activity and prevention of TRPML1 degradation (28). Decreased mTOR activity means that TFEB is no longer kept in the cytosol by phosphorylation but tanslocates into the nucleus (29), where it promotes the transcription of genes encoding lysosomal proteins and the kinase ULK1 as inducer of autophagy. In addition, TRPML1 contributes to the regulation of TFEB through the protein phosphatase calcineurin. Both, enhanced lysosomal biogenesis and induction of autophagy promote the ability of cathepsin B deficient macrophages to get rid of ingested bacteria. TRPML3 has also been shown by several groups to be involved in autophagy (30–32), suggesting that as described above for TRPML1 it may play similar roles in certain cell types. Evidence for an evolutionary conserved role of TRPMLs in bacterial clearance by macrophages comes from drosophila work. Thus, Wong et al. (33) found that flies lacking trpml in macrophages exhibited compromised clearance of *E. coli*, a phenotype similar to the one observed in macrophages deficient of ClC-b, the drosophila homolog of the mammalian late-endosomal/lysosomal Cl^−^/H^+^ transporter CLCN7. Wong et al. further showed that ClC-b-mediated Cl^−^ transport into endolysosomes was necessary for the accumulation of luminal Ca^2+^, which, when released through TRPML, drives the delivery of phagocytic cargo to lysosomes for degradation. Beside phagocytosis, macrophages play another important role within the innate immune system as they produce and secrete a variety of cytokines and chemokines after stimulation. Inflammatory cytokines and chemokines are signaling molecules that attract other immune cells to the inflammation herd. Apart from this, cytokines may also determine the polarization state of macrophages, where classically activated macrophages (M1) are stimulated by interferon-γ, TLR ligands, or microbial substrates like LPS. This is connected with an increased production of proinflammatory cytokines, such as TNF-α, IL-6, IL-1, IL-23 and reactive oxygen/nitrogen species. These macrophages have a high pro-inflammatory and microbicidal activity. Alternatively, activated macrophages (M2) are stimulated by IL-4 and IL-13 and are involved in tissue repair, suppression of inflammation and tumor progression by secreting the anti-inflammatory cytokines IL-10 and TGF-β. A third class of macrophages are regarded as tumor-associated macrophages. They differentiate from circulating monocytes after migrating into tumor tissue upon stimulation with IL-4, IL-10, or IL-13 and exhibit pro-tumorigenic functions (34, 35).

The release of cytokines by macrophages is a highly regulated process. Time, volume and site of release must be controlled, but first cytokines need to be transported to the plasma membrane. To this end, they make use of the cell's trafficking machinery including the trans-Golgi network (TGN) and the endolysosomal system, especially recycling endosomes (36). In the latter ones high levels of TRPML2 are found (37, 38). Sun et al. demonstrated that lack of TRPML2 in BMDM leads to an intracellular accumulation and decreased secretion of CCL2 [chemokine (C-C motif) ligand 2, also called monocyte chemoattractant protein 1 (MCP1)] (20). They also found reduced macrophage recruitment in TRPML2^−/−^ mice after LPS stimulation which is in accordance with the role of CCL2 as chemoattractant recruiting additional immune cells to the site of inflammation (39). The link between TRPML2 and CCL2 was further investigated by Plesch et al. who developed ML2-SA1, a selective agonist for TRPML2, and found that activation of the channel directly stimulates the secretion of CCL2 from BMDM. The release is most likely mediated via the early/recycling endosomal pathway, since activation of TRPML2 by ML2-SA1 promotes trafficking through this pathway. In addition, Plesch et al. found that direct activation of TRPML2 leads to enhanced recruitment of macrophages (5). These findings by Sun et al. and Plesch et al. strongly suggest that TRPML2 plays a crucial role in the release of CCL2 and likely other chemokines as well as in the stimulation of macrophage migration.

### Neutrophils

Neutrophils are another essential cell type in the innate immune system. They are the first cells to arrive at the site of inflammation or infection as they have a high chemotactic ability (40). They carry out numerous functions: First, they express and secrete cytokines that recruit more immune cells like macrophages to amplify the inflammatory response (41). Second, they perform phagocytosis of pathogens resulting in phagosome formation and fusion with lysosomes to kill engulfed bacteria (41, 42). Third, they release a variety of antimicrobial proteins (cathepsins, neutrophil elastase, lysozyme, NADPH oxidase) that help eliminate the pathogens (41).

In 2017, Dayam et al. showed the lipid kinase PIKfyve to play an essential role in coordinating various neutrophil functions. As mentioned above, PIKfyve is responsible for the synthesis of PI(3,5)P_2_, an endogenous activator of TRPML channels that results in lysosomal Ca^2+^ release also in neutrophils. The authors further found that the PIKfyve-TRPML1-Ca^2+^ axis regulates phagosome maturation, i.e., the fusion of phagosomes and lysosomes. They also found that inhibition of this axis blocks phagosome maturation (43). These findings are in line with their previous works and other works by Kim et al. (22), reporting on the same PIKfyve-TRPML1-Ca^2+^ pathway to trigger phagosome-lysosome-fusion in macrophages (21, 22). Furthermore, Dayam et al. found that PIKfyve activity is essential for ROS generation and chemotaxis mediated through the stimulation of Rac GTPases. Taken together these data suggest that PIKfyve and TRPML1 are important regulators of several neutrophil functions that are critical for the rapid response of the innate immune system (43).

### Dendritic Cells

Dendritic cells (DCs) are antigen-presenting cells (APCs) which play key roles in the adaptive immune response (44, 45). After capturing pathogenic antigens via macropinocytosis, immature DCs turn into mature DCs. They start to process and express high level of antigens, stimulatory molecules, and cytokines that are able to induce the T-cell response after migrating to lymph nodes, where they present the antigens to activate T-cells (46–48). Intracellular Ca^2+^ signaling from endolysosomal TRPML1 channel is involved in DC functions, such as regulating TLRs for nucleic acid sensing and DC migration (18). TLRs can recognize structurally conserved molecules from pathogens. Membrane lipids from pathogens can be recognized by cell surface located TLR1, TLR2, TLR4, and TLR6 while nucleic acids from pathogens can be recognized by intracellular located TLR3, TLR7, TLR8, and TLR9 (49–51). TRPML1 has been reported to be involved in the TLR7 response to single strand RNA (ssRNA). Li et al. demonstrated that loss of TRPML1 function or inhibition of PI(3,5)P_2_ generating PIKfyve blocks the transportation of ssRNA into lysosomes while activation of TRPML1 by the TRPML channel agonist ML-SA1 enhances this process. Impaired transportation of ssRNA leads to an impaired TLR7 response, demonstrating that the P(3,5)P_2_-TRPML1 axis plays an important role in this process (52).

Regarding the role of TRPML1 in DC migration, Bretou et al. (18) found that after the down regulation of macropinocytosis in DCs upon sensing the pathogens, lysosomal calcium signaling through TRPML1 regulates DC chemotaxis and migration to lymph nodes by controlling the motor protein myosin II retrograde flow at the cell rear to induce fast and directional migration. TRPML1 mediated calcium signaling further initiates TFEB translocation from cytoplasm to nucleus which will further maintain TRPML1 expression forming a positive feedback loop mediated by the TFEB-TRPML1 axis. Therefore, activation of the TFEB-TRPML1 axis via the inhibition of macropinocytosis is a critical step to switch DCs from patrolling mode to fast migration mode and homing into lymph nodes (18).

### Natural Killer Cells

Natural killer (NK) cells differentiate from the same lymphoid progenitor as T- and B-cells but are classified as innate immunity lymphocytes because of their rapid response to pathogens, especially viruses and fungi. NK cells are also functionally active against tumor cells (53, 54). Killing of target cells is mediated by cytotoxic factors, such as perforin and granzymes which are secreted from lysosome related organelles called lytic granules in NK cells (55, 56). Besides killing target cells directly through cytotoxicity, NK cells can indirectly contribute to immune defense via secretion of cytokines, such as interferon-γ (IFN-γ) or tumor necrosis factor–α (TNF-α) to regulate antigen-presenting cell function and T cell responses (57). NK cell activity is regulated by the dynamic balance between activating and inhibitory signals generated from a combination of germ-line encoded receptors which recognize the ligands expressed on the target cell surface. These determine whether or not the NK cell will kill the target cell (58, 59). Major histocompatibility complex (MHC) class I molecules are antigen-presenting molecules and critical for adaptive immune responses, which can be recognized by inhibitory receptors, such as killer cell Ig-like receptors (KIR), Ly49, and CD94/NKG2A on NK cells (60, 61). Decrease of MHC class I expression occurs when cells are infected by virus or under cellular transformation. This situation is called “missing-self” and can be detected by NK cells and promote NK cell cytotoxicity and cytokine production to selectively kill the target cell (57, 62). A process called NK cell education is the interaction between self-MHC and inhibitory receptors on NK cells to calibrate NK cell effector capacities (63). Goodridge et al. have recently reported that TRPML1 is involved in this process by modulating secretory lysosomes, granzyme B content, and by regulating effector function in NK cells (19). Goodridge et al. found that silencing of TRPML1 or pharmacological interference with PIKfyve resulted in enlarged lysosomes with increased granzyme B content and higher effector function, representing the educated state of NK cells. Therefore, these findings establish a link between NK cell education and remodeling of the lysosomal compartment. They also suggest a potential way to increase NK cell function via manipulating calcium homeostasis within lysosome related organelles (19).

## Chapter II: Adaptive Immune System

### B Cells

B cells form a vital part of the adaptive immune system. They cooperate with other immune cells, such as T-cells, macrophages, and dendritic cells to eliminate foreign antigens. B-cells operate by producing and secreting millions of different antibody molecules, which in turn recognize and respond to a pathogen or foreign antigen. This response is partially mediated by an integral membrane protein called the B-cell receptor (BCR) (64, 65). BCRs are specialized receptors, structurally composed of two Ig light chains, two Ig heavy chains, and two heterodimers Igα and Igβ (65). B-cells have a specialized lysosomal compartment in which antigens deriving from endocytosed BCRs are loaded onto MHC class II. Upon BCR engagement, this compartment undergoes a regulated transformation linked to the *de-novo* formation of multi-vesicular bodies (MVBs) which mature from tubulo-vacuolar early endosomes by a process of remodeling (66, 67). Although these processes are thought to be crucial for the role of B-lymphocytes in immune-modulatory and antigen processing, the molecular pathways underlying the regulation and formation of the specialized lysosomal compartment within B-cells are still poorly understood (66). Specifically, the distribution of MHC class II products over endolysosomal compartments including MVBs in response to BCR engagement remains a matter of debate (68).

Song et al. (66) have verified the expression of TRPML1 and TRPML2 in B-lymphocytes. Their results however indicate that TPRML1 deficient B-lymphocytes are not linked to gross changes in the lysosomal compartment. This is in accordance with the finding that MLIV patients do not exhibit any obvious abnormalities in lymphocyte function, nor do they have obvious immune function defects, arguing for compensatory mechanisms. The authors suggested that normal lysosomal compartments seen in lymphocytes with TRPML1 deficiency may be due to a role of TRPML2 in compensating for the loss of TRPML1 function, and therefore postulated overlapping functions of TRPML1 and TRPML2 in B-lymphocytes. In contrast, expression of TRPML3 had not been demonstrated for B cells (66).

Normal immune response is dependent on an intact development of B-lymphocytes and mutations of genes involved in B-cell differentiation distort this process. Bruton's tyrosine kinase (Btk) gene, that is part of the Tec family of cytoplasmic non-receptor protein-tyrosine kinases, encodes for one of these crucial molecules. Mutations in this gene lead to a partial blockage between the pre- and pro-B-lymphocyte stage, and a complete blockage between the pre- and mature B-lymphocyte stage, leading to X-linked immunodeficiency pathologies in mice and to X-linked agammaglobulinemia in humans. A more severe phenotype is observed in humans as compared to mice because only a partial blockage is established between the pre- and mature B-cell stage in the latter (69).

The phosphorylation and activation of Btk is dependent on plasma membrane (PM) localization. BCR engagement by an antigen results in the activation of PI3K, generating the phosphoinositide phosphatidylinositol-3,4,5-trisphosphate (PIP3). Btk, along with other signaling proteins, are then recruited, as a result of PIP3 accumulation to the PM. Subsequent binding of the pleckstrin homology (PH) domain to PIP3 is a pre-requisite for Btk activation. Even though the molecular mechanisms which target Btk to the cell surface remain largely unclear, the PH domain seems to play an important role. As Btk is a cytoplasmic tyrosinase kinase, loss of Btk leads to dysregulation in downstream signaling pathways, impacting several effector molecules (70).

Lindvall et al. have shown that TRPML2 is expressed in T-, B-, myeloma, and mastocytoma cell lines, in addition to whole primary splenocytes. TRPML2 was also shown to be expressed at pre-B cell, mature B-cell, and plasma cell stages, and in splenic T1 B-lymphocyte cell populations. TRPML2 was up-regulated in both Btk-defective and wild-type splenic primary mouse B-cells post-stimulation with either phorbol-12-myristate-13-acetate (PMA) plus ionomycin or anti-IgM while it was downregulated by a factor of four in unstimulated Btk-defective splenic primary mouse B-cells. The authors further proposed a role of Btk in B-cells in suppressing the activation of TRPML2. However, these results await further confirmation.

## Conclusions and Outlook

**Figure 3:**
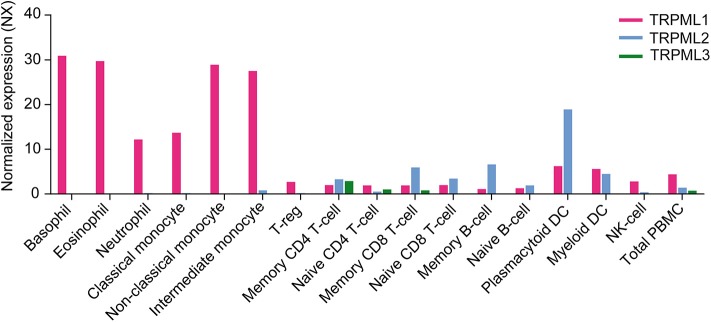
Gene expression level in various human immune cells showing the RNA sequencing data averaged from three different sources (Internally generated Human Protein Atlas (HPA) as well as data generated by Monaco et al. and Schmiedel et al.) Data are shown as normalized expression (NX) (71, 72), resulting from an internal normalization pipeline for the different cell types and total peripheral blood mononuclear cells (PBMC). The NX value for each gene represents the maximum NX value in the three data sources (Based on data from Human Protein Atlas: Blood Cell Type Expression (RNA) of MCOLN1/MCOLN2/MCOLN3 available from: https://www.proteinatlas.org).

Functional expression of TRPML channels has been demonstrated for a number of cells belonging to both the innate and the adaptive immune system, in particular macrophages, dendritic cells, neutrophils, NK cells, and B lymphocytes as outlined above. Based on gene expression data from different sources (Figure 3), TRPML channels may however be functionally active in many more cells of the immune system, such as basophils, eosinophils, monocytes as well as CD4+ and CD8+ T cells. Which functional roles they have in these very diverse immune cell types remains to be elucidated as well as potential functional differences between the different TRPML channels which may act as homomers or heteromers in some of these cells.

## Author Contributions

All authors wrote and discussed the manuscript. Y-KC and C-CC provided the patch-clamp data in Figure 1.

### Conflict of Interest

The authors declare that the research was conducted in the absence of any commercial or financial relationships that could be construed as a potential conflict of interest.
